# An efficient framework for protein-protein interaction prediction by integrating stacked denoising autoencoders and random ferns

**DOI:** 10.1016/j.isci.2026.115100

**Published:** 2026-02-20

**Authors:** Zheng Wang, Lei Wang, Yang Li, Zhu-Hong You, Yue-Chao Li

**Affiliations:** 1 School of Computer Science and Engineering, Xi’an University of Technology, Xi’an 710048, China; 2 School of Mathematics and Statistics, Weinan Normal University, Weinan 714099, China; 3 School of Computer Science, Northwestern Polytechnical University, Xi’an 710072, China

**Keywords:** Biochemistry, Structural biology, Biocomputational method

## Abstract

Protein-protein interactions (PPIs) are crucial for understanding disease and discovering drug targets. To overcome the limitations of experimental methods, we propose SDAERFs, a computational framework that predicts PPIs from protein sequences. It leverages evolutionary information in position-specific scoring matrices (PSSMs), employs a stacked denoising autoencoder (SDAE) for feature extraction, and uses a Random Ferns (RFs) classifier for prediction. Extensive validation on benchmark datasets yielded high accuracies of 98.13% and 98.60%. Comprehensive comparisons confirmed the model’s superior performance. SDAERFs provides an efficient and reliable tool for advancing PPI prediction and therapeutic development.

## Highlights

• Achieves over 98% accuracy using only protein sequence information
• Integrates stacked denoising autoencoders and random ferns for robust interaction prediction
• Validates strong cross-species generalization capability via independent benchmark testing
• Provides a computationally efficient framework suitable for high-throughput screening

## Graphical abstract

**Figure:**
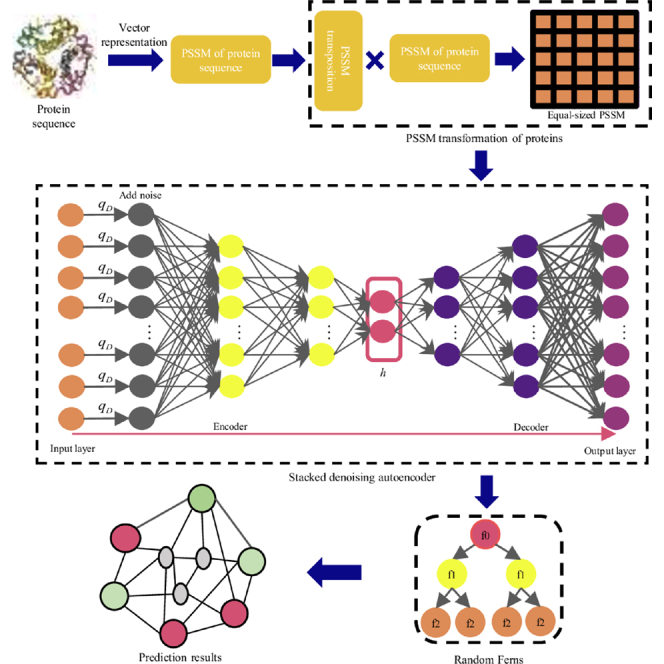

## Introduction

Protein-protein interactions (PPIs) are fundamental to various cellular processes, including DNA synthesis, immune responses, gene transcription, and signal transduction.^1^ Understanding these interactions is crucial for elucidating protein functions, identifying functional modules, determining disease mechanisms, and developing therapeutic strategies.^2^^,^^3^ Despite their importance, traditional experimental methods for identifying PPIs, such as mass spectrometry,^4^ metabolic regulation,^5^ tandem affinity purification,^6^ therapeutic targets for Alzheimer’s disease,^7^ and yeast two-hybrid assays,^8^ are often time-consuming, costly, and prone to high rates of false positives and false negatives. These limitations have necessitated the development of computational methods to predict PPIs efficiently and accurately.^9^

The swift advancement of genome sequencing technologies has resulted in an exponential growth in protein sequence data, further highlighting the potential of sequence-based methods for PPIs prediction. Machine learning computational methods, such as support vector machines,^10^ random forests,^11^ and neural networks,^12^ have been widely adopted for this purpose. For example, Zeng et al.^13^ proposed a deep learning framework for predicting PPIs, integrating both local contextual and global sequence features. Utilizing a sliding window approach, it captured local features, while a convolutional neural network extracted global characteristics from entire protein sequences. Hashemifar et al.^14^ introduced a DPPI algorithm for predicting PPIs using sequence data. This method combines a Siamese-like CNN and random projection techniques, significantly improving prediction accuracy. Li et al.^15^ developed an efficient ensemble classifier for predicting PPIs by integrating PSSM information with a Local Binary Pattern feature extraction method. This approach employs a Rotation Forest classifier to enhance predictive accuracy. Despite significant advancements in computational PPI prediction, several challenges remain. Many existing methods rely on multiple feature extraction techniques, which can introduce redundancy and noise, complicating model development. Thus, creating models that can effectively utilize sequence information without extensive feature engineering remains an open challenge.^16^

Inspired by these challenges, this article presents a computational framework, termed SDAERFs, for identifying PPIs. Our model leverages evolutionary information encoded in PSSM matrices, which capture the positional specificity of amino acid substitutions in protein sequences. Distinct from prior works that combine CNNs with random projection (Hashemifar et al.^14^) or ensemble local feature extractors with classifiers such as Rotation Forest (Li et al.^15^), our framework introduces two key methodological innovations. First, we employ a Stacked Denoising Autoencoder (SDAE) to learn robust, high-level feature representations directly from PSSMs. Compared to standard deep learning architectures, the unsupervised pre-training and denoising mechanism of SDAE are particularly suited for handling the sparse and noisy nature of evolutionary matrices, promoting the extraction of more generalizable features. Second, we adopt Random Ferns (RFs) as the classifier. RFs provide a highly efficient and regularized alternative to complex ensembles or deep classifiers, effectively mitigating overfitting in high-dimensional feature spaces—a property we later validate through superior cross-species generalization performance. The Stacked Denoising Autoencoder (SDAE) is employed to extract high-level features from PSSM matrices, while the Random Ferns (RFs) classifier is used to predict PPIs based on these features. The effectiveness of the SDAERFs model was validated on Yeast and Human datasets, where it achieved high prediction accuracy. Comparative experiments with existing methods demonstrated the superior performance of our model in terms of accuracy, sensitivity, and MCC. These results highlight the reliability and robustness of SDAERFs for PPIs prediction. The SDAERFs model represents an important tool for the computational prediction of PPIs, offering a robust and efficient approach for identifying protein interactions.

## Results

### Prediction performance on two protein-protein interaction datasets

**Figure 1:**
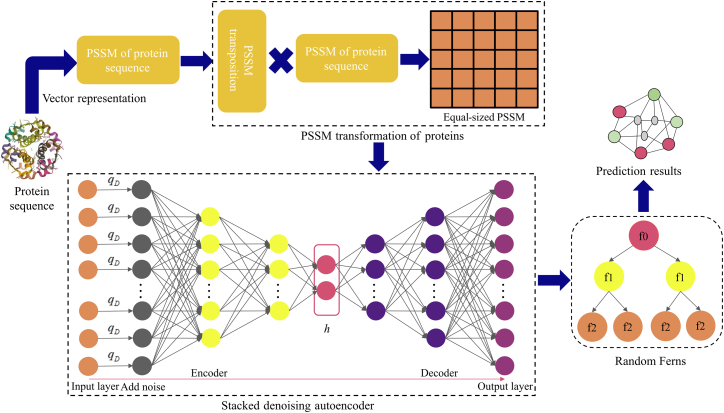
The flowchart of the SDAERFs method

**Figure 2:**
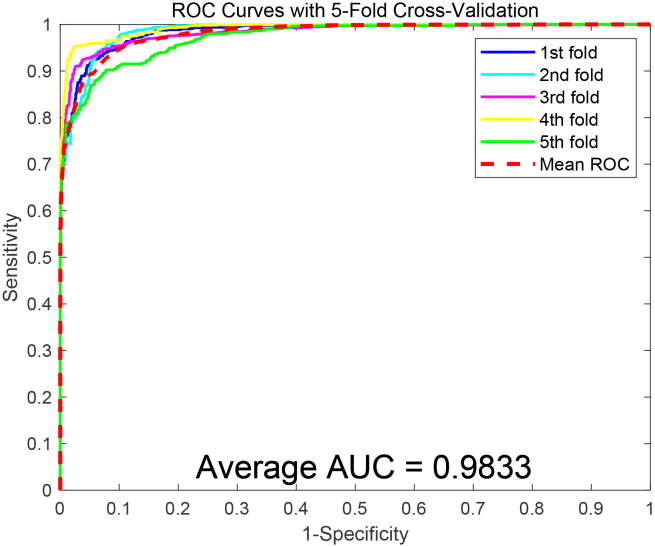
ROC curves obtained for SDAERFs on the Yeast dataset

**Figure 3:**
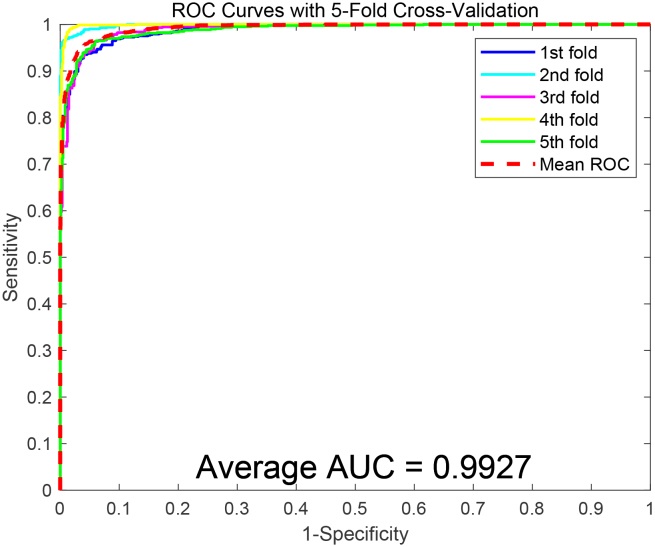
ROC curves obtained for SDAERFs on the Human dataset

To assess the predictive capability of the SDAERFs, which combines StackedDAE, PSSM, and RFs for PPIs identification, we performed an in-depth analysis using two benchmark datasets: Yeast and Human. The flowchart of SDAERFs is shown in Figure 1. As shown in Table 1, the experimental results on the Yeast dataset demonstrated the robust performance of the SDAERFs model. The average accuracy, precision, sensitivity, and MCC values were 98.13%, 98.57%, 97.66%, and 96.27%, respectively, with standard deviations of 0.91%, 0.32%, 1.64%, and 1.80%. The ROC curve for the Yeast dataset is shown in Figure 2, with an AUC value of 98.33%, indicating strong predictive power. On the Human dataset, the SDAERFs model achieved even higher performance metrics, which are represented in Table 2. The average accuracy, precision, sensitivity, and MCC values were 98.60%, 98.11%, 98.99%, and 97.21%, respectively, with standard deviations of 0.56%, 1.21%, 0.50%, and 1.09%. The ROC curve for the Human dataset is shown in Figure 3, with an AUC value of 99.27%, further highlighting the model’s excellent predictive capabilities.

**Table 1:** Experimental performance of SDAERFs on the Yeast dataset

Testing Set	ACC (%)	PE (%)	SN (%)	MCC (%)	AUC (%)
1	98.08	98.84	97.35	96.17	98.42
2	98.66	98.78	98.61	97.32	98.55
3	97.86	98.34	97.27	95.71	98.30
4	99.24	98.76	99.73	98.48	99.17
5	96.83	98.12	95.35	93.68	97.22
Average	98.13 ± 0.91	98.57 ± 0.32	97.66 ± 1.64	96.27 ± 1.80	98.33 ± 0.70

The average values are presented as mean ± standard deviation, calculated from the five testing sets.

**Table 2:** Experimental performance of SDAERFs on the Human dataset

Testing Set	ACC (%)	PE (%)	SN (%)	MCC (%)	AUC (%)
1	98.35	98.31	98.19	96.68	98.90
2	99.14	99.03	99.27	98.28	99.83
3	98.65	97.99	99.05	97.29	98.92
4	99.08	99.09	98.96	98.16	99.89
5	97.79	96.12	99.50	95.65	98.80
Average	98.60 ± 0.56	98.11 ± 1.21	98.99 ± 0.50	97.21 ± 1.09	99.27 ± 0.54

The average values are presented as mean ± standard deviation, calculated from the five testing sets.

These results demonstrate that the integration of PSSM, StackedDAE, and RFs within the SDAERFs model effectively captures the complex patterns in PPIs data, leading to accurate and reliable predictions. The combination of evolutionary information from PSSM, robust feature extraction via StackedDAE, and the reliable classification ability of RFs contributes to the model’s superior performance. The low standard deviations across all metrics and datasets underscore the model’s stability and consistency, making it a reliable tool for predicting PPIs in both Yeast and Human datasets.

### Comparison with and without stacked denoising autoencoder feature embedding

To validate the effectiveness of the stacked denoising autoencoder feature embedding in the SDAERFs model, we conducted a comparative experiment by evaluating the model’s performance with and without the SDAE component. This analysis aimed to demonstrate the contribution of the SDAE in enhancing the model’s predictive accuracy and feature representation capabilities. To assess the impact of the SDAE, we compared the model’s performance on two PPI datasets under two scenarios: with SDAE feature embedding (SDAERFs) and without it (comparison model). Table 3 summarizes the comparison results for the Human and Yeast datasets. In comparison, the model without SDAE on the Yeast dataset achieved an accuracy of 73.07%, a PE of 75.82%, an SN of 67.85%, and an MCC of 46.47%, which were significantly lower than those of the proposed model. Similarly, the comparison model on the Human dataset is also significantly lower than the proposed model on all indicators. These results conclusively demonstrate that the integration of the SDAE significantly enhances the feature representation and the model’s ability to make accurate predictions. Therefore, incorporating the SDAE component is crucial for the accuracy and reliability of SDAERFs in predicting protein-protein interactions.

**Table 3:** Performance comparison with and without the SDAE feature embedding model

Datasets	Method	ACC (%)	PE (%)	SN (%)	MCC (%)	AUC (%)
Yeast	comparison model	73.07 ± 0.63	75.82 ± 1.54	67.85 ± 2.52	46.47 ± 1.03	52.30 ± 0.99
	SDAERFs	98.13 ± 0.91	98.57 ± 0.32	97.66 ± 1.64	96.27 ± 1.80	98.33 ± 0.70
Human	comparison model	76.79 ± 0.44	77.16 ± 0.74	73.05 ± 1.47	53.46 ± 0.77	53.51 ± 0.56
	SDAERFs	98.60 ± 0.56	98.11 ± 1.21	98.99 ± 0.50	97.21 ± 1.09	99.27 ± 0.54

Data are presented as mean ± standard deviation.

### Comparison of different feature representations

To assess the impact of SDAE feature extraction in SDAERFs, we performed a comparative study using different feature representation techniques on the Yeast and Human datasets. This section compared the performance of the SDAERFs model, which employs SDAE for feature extraction, with a comparison model that uses principal component analysis (PCA) for feature dimensionality reduction.^17^ This comparison aimed to demonstrate the superiority of the SDAE in capturing complex and discriminative features from the PSSM of protein sequences. The results of the comparison are summarized in Tables 4 and 5 for the Human and Yeast datasets, respectively. In contrast, the comparison model with PCA on the Human dataset achieved an ACC of 92.07%, a PE of 92.45%, an SN of 90.87%, and an MCC of 84.13%. The standard deviations for the PCA model were consistently higher, indicating less stability in its performance. On the other hand, the PCA model on the Yeast dataset achieved an ACC of 89.56%, a PE of 93.99%, an SN of 84.74%, and an MCC of 79.78%. The standard deviations for the PCA model were again higher, underscoring the enhanced robustness of the SDAERFs.

**Table 4:** Performance comparison of the SDAERF model with the PCA feature model on the Human dataset

Testing Set	ACC (%)	PE (%)	SN (%)	MCC (%)	AUC (%)
1	92.59	94.39	90.58	85.25	79.05
2	94.55	96.42	92.14	89.15	80.45
3	93.57	94.06	91.95	87.07	80.34
4	90.38	88.65	90.22	80.61	76.90
5	89.28	88.71	89.49	78.55	78.04
Average	92.07 ± 2.20	92.45 ± 3.55	90.87 ± 1.14	84.13 ± 4.43	78.96 ± 1.52
SDAERFs	98.60 ± 0.56	98.11 ± 1.21	98.99 ± 0.50	97.21 ± 1.09	99.27 ± 0.54

Data for the “Average” and “SDAERFs” rows are presented as mean ± standard deviation, calculated from the five testing sets.

**Table 5:** Performance comparison of the SDAERF model with the PCA feature model on the Yeast dataset

Testing Set	ACC (%)	PE (%)	SN (%)	MCC (%)	AUC (%)
1	90.44	89.49	92.15	80.89	83.30
2	86.28	94.24	76.51	73.68	83.86
3	92.31	91.67	93.44	84.63	85.78
4	88.60	96.48	79.71	78.28	86.87
5	90.17	98.07	81.90	81.43	88.66
Average	89.56 ± 2.26	93.99 ± 3.48	84.74 ± 7.61	79.78 ± 4.09	85.69 ± 2.20
SDAERFs	98.13 ± 0.91	98.57 ± 0.32	97.66 ± 1.64	96.27 ± 1.80	98.33 ± 0.70

Data for the “Average” and “SDAERFs” rows are presented as mean ± standard deviation, calculated from the five testing sets.

**Figure 4:**
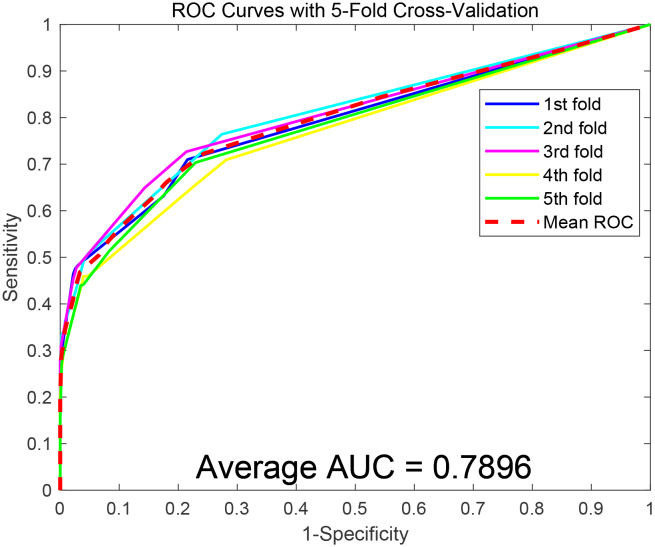
ROC curves obtained using the PCA feature extraction method on the Human dataset

**Figure 5:**
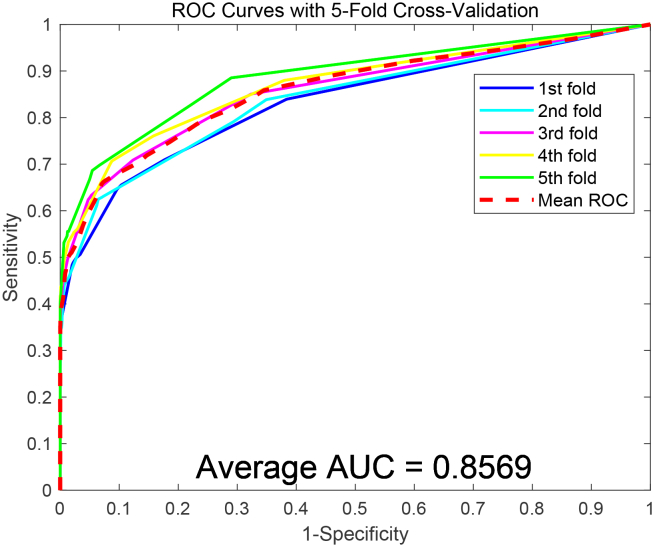
ROC curves obtained using the PCA feature extraction method on the Yeast dataset

The ROC curves based on the PCA model are presented in Figures 4 and 5. In contrast, the PCA model achieved AUC values of 78.96% and 85.69% on the two datasets, respectively, further highlighting the improved predictive power of the SDAERFs model. The comparative analysis highlights that the SDAE-based feature extraction method significantly outperforms the PCA-based approach in terms of accuracy, precision, sensitivity, MCC, and AUC. The superior performance of the SDAERFs model can be attributed to the SDAE’s ability to extract robust and discriminative features from the PSSM, leading to enhanced predictive performance and reliability. This underscores the significance of employing advanced feature extraction to enhance the precision of PPIs recognition models.

### Compared with different classifier models

To assess the performance of the Random Ferns classifier within the SDAERFs model, we conducted a comparative study with several other classification algorithms on the Human dataset. Specifically, we compared the experimental results of the SDAERFs model, which employs RFs as the classifier, with models using K-Nearest Neighbor (KNN), Support Vector Machine (SVM), Naive Bayes (NB), Decision Tree (DT), Random Forest (RF), and Neural Network (NN). The goal of this comparison was to highlight the RF classifier’s superior performance in detecting PPIs when applied to features extracted using a stacked denoising autoencoder. For the comparative analysis, we maintained the same SDAE-based feature extraction method across all models, ensuring that the differences in performance were solely due to the choice of classifier.

Table 6 summarizes the results of the comparison of different classifier models on the Human dataset. The SDAERFs model performs outstandingly, especially in the three metrics of ACC, PE, and MCC, and its average prediction results outperform those of the SVM-based, DT-based, NB-based, and KNN-based methods. The comparative analysis confirms that the SDAERFs method with the RFs classifier significantly outperforms models that use SVM, DT, NB, RF, NN, and KNN. The superior performance of the SDAERFs model can be attributed to the RFs classifier’s ability to effectively handle the complex and high-dimensional feature space derived from the SDAE. This underscores the crucial role of selecting suitable classification algorithms to enhance the effectiveness and reliability of PPIs detection methods.

**Table 6:** Performance comparison of SDAERFs with different classifier models on the Human dataset

Models	ACC (%)	PE (%)	MCC (%)	AUC (%)
SDAERFs Model	98.60 ± 0.56	98.11 ± 1.21	97.21 ± 1.09	99.27 ± 0.54
SVM Model	95.69 ± 0.73	91.90 ± 1.50	91.69 ± 1.36	99.95 ± 0.03
DT Model	94.11 ± 1.83	91.70 ± 1.87	88.32 ± 3.64	94.19 ± 1.89
NB Model	96.96 ± 0.42	94.00 ± 0.98	94.10 ± 0.79	98.46 ± 0.16
KNN Model	97.41 ± 0.41	94.88 ± 0.89	94.95 ± 0.80	99.96 ± 0.02
RF Model	97.84 ± 1.56	97.27 ± 3.03	95.74 ± 3.03	97.87 ± 1.51
NN Model	97.15 ± 1.17	97.44% ± 3.01%	94.43 ± 2.23	97.77 ± 1.57

Data are presented as mean ± standard deviation.

### Compared with existing methods

We performed a comprehensive comparison of the SDAERFs in predicting PPIs, using the Yeast and Human datasets, against several established methods to assess their performance. These methods include various feature extraction and classification techniques widely used in the field of PPIs prediction. For consistency and fairness, the comparison was carried out using the same 5-fold cross-validation technique. The SDAERFs model, which combines a stacked denoising autoencoder for feature extraction and Random Ferns for classification, was compared against various advanced methods, including models based on different feature representation techniques and various classification algorithms. The evaluation metrics used for comparison include ACC, PE, SN, and MCC. Tables 7 and 8 summarize the results of the comparison of SDAERFs with existing models on two benchmark datasets, respectively.

**Table 7:** Comparative results of existing methods on the Yeast dataset

Models	ACC	PE	SN	MCC
SDAERFs	0.9813 ± 0.0091	0.9857 ± 0.0032	0.9766 ± 0.0164	0.9627 ± 0.0180
MARPPI (Li et al.^18^)	0.9603 ± 0.0076	0.9812 ± 0.0098	0.9351 ± 0.0122	0.9183 ± 0.0132
MatFLDA_RFs (Li et al.^19^)	0.9503 ± 0.0025	0.9914 ± 0.0026	0.9084 ± 0.0047	0.9052 ± 0.0045
DeepFE-PPI (Yao et al.^20^)	0.9478 ± 0.0061	0.9645 ± 0.0087	0.9299 ± 0.0066	0.8962 ± 0.0123
DeepPPI (Du et al.^21^)	0.9443 ± 0.0030	0.9665 ± 0.0059	0.9206 ± 0.0036	0.8897 ± 0.0062
OLPP-RoF (Li et al.^22^)	0.9007 ± 0.0060	0.9024 ± 0.0056	0.8983 ± 0.0141	0.8210 ± 0.0097

Data are presented as mean ± standard deviation.

**Table 8:** Comparative results of existing methods on the Human dataset

Models	ACC	PE	SN	MCC
SDAERFs	0.9860 ± 0.0056	0.9811 ± 0.0121	0.9899 ± 0.0050	0.9721 ± 0.0109
SIFT-WELM (Li et al.^23^)	0.9760 ± 0.0057	0.9622 ± 0.0106	0.9894 ± 0.0029	0.9523 ± 0.0112
LPQ-RoF (Wong et al.^24^)	0.9796 ± 0.0022	0.9835 ± 0.0061	0.9732 ± 0.0073	0.9600 ± 0.0040
RPEC (Song et al.^25^)	0.9659 ± 0.0124	0.9618 ± 0.0117	0.9672 ± 0.0141	0.9318 ± 0.0249
GARVM (An et al.^26^)	0.9303 ± 0.0088	0.9473 ± 0.0213	0.9059 ± 0.0120	0.8612 ± 0.0145
GSRVM (An et al.^26^)	0.9215 ± 0.0120	0.9108 ± 0.0079	0.9178 ± 0.0152	0.8545 ± 0.0235
PSORVM (An et al.^26^)	0.9350 ± 0.0090	0.9640 ± 0.0138	0.9191 ± 0.0112	0.8802 ± 0.0103
GWORVM (An et al.^26^)	0.9456 ± 0.0052	0.9308 ± 0.0109	0.9555 ± 0.0091	0.8951 ± 0.0114
OLPP-RoF (Li et al.^27^)	0.9609 ± 0.0024	0.9656 ± 0.0036	0.9520 ± 0.0034	0.9247 ± 0.0046

Data are presented as mean ± standard deviation.

As described in Table 7, the SDAERFs model achieved an average ACC of 98.13%, a sensitivity of 97.66%, and an MCC of 96.27% on the Yeast dataset. In comparison, the existing methods showed varying degrees of performance, with the best-performing model achieving an accuracy of 96.03%. The MCC value of the SDAERFs model was particularly notable, significantly exceeding those of the competing models. On the Human dataset, SDAERFs demonstrated exceptional performance, achieving an average ACC of 98.60%, a PE of 98.11%, an SN of 98.99%, and an MCC of 97.21%. The existing methods on this dataset showed average accuracies ranging from 92.15% to 97.96%, with the SDAERFs model surpassing all of them. The SDAERFs model consistently outperformed all other methods based on both MCC and ACC evaluation indicators, demonstrating its superior ability to predict PPIs accurately. The comparative analysis demonstrates the superior performance of the SDAERFs model over existing methods in detecting PPIs across the two standard datasets.

### Cross-species generalization analysis

To assess the model’s ability to generalize beyond its training data, we conducted an independent validation experiment. The SDAERFs framework was trained on the complete Yeast dataset and subsequently tested on the complete and independent Human dataset. This strict separation between training and testing data provides a realistic evaluation of performance on a distinct, unseen organism. The cross-species prediction results are detailed in Table 9. The SDAERFs model (SDAE + Random Ferns) achieved a cross-species accuracy (ACC) of 59.24%. While this is a substantial decrease from its exceptional within-species performance (>98%), it remains significantly above the random baseline of 50.00%. This result confirms that the model successfully captures a meaningful subset of evolutionarily conserved patterns that are generalizable across species boundaries.

**Table 9:** Cross-species independent validation performance (Training: Full Yeast dataset; Testing: Full Human dataset)

Models	ACC	PE	SN	MCC
SDAERFs (RFs)	0.5924	0.5514	0.7876	0.2162
SVM	0.5272	0.7808	0.0146	0.0576
RF	0.3780	0.3948	0.5668	−0.2452
BP	0.5030	0.3987	0.08147	−0.0516
*Random Baseline*	0.5000	–	–	0.0000

An analysis of Table 9 reveals critical insights into classifier robustness for cross-domain prediction. While the SDAERFs model maintains a balanced and above-chance performance across all metrics, other classifiers trained on the same SDAE features exhibit severe limitations. The Support Vector Machine (SVM), for instance, shows a high precision (PE = 0.7808) but an extremely low sensitivity (SN = 0.0146), indicating a strong bias toward predicting negative instances, which is unsuitable for practical use. Both the Random Forest (RF) and the neural network (BP) classifier perform at or below the random baseline, with negative MCC values signifying predictions worse than random guessing. This “negative transfer” phenomenon suggests that these more complex models heavily overfit to species-specific noise in the source (Yeast) data, which becomes detrimental when applied to the target (Human) distribution. The superior performance of the Random Ferns classifier in this challenging setting validates a key design choice of the SDAERFs framework. Its simpler, more regularized structure appears to discourage overfitting to idiosyncratic features and instead promotes the learning of more generalizable, evolutionarily stable interaction signatures.

**Figure 6:**
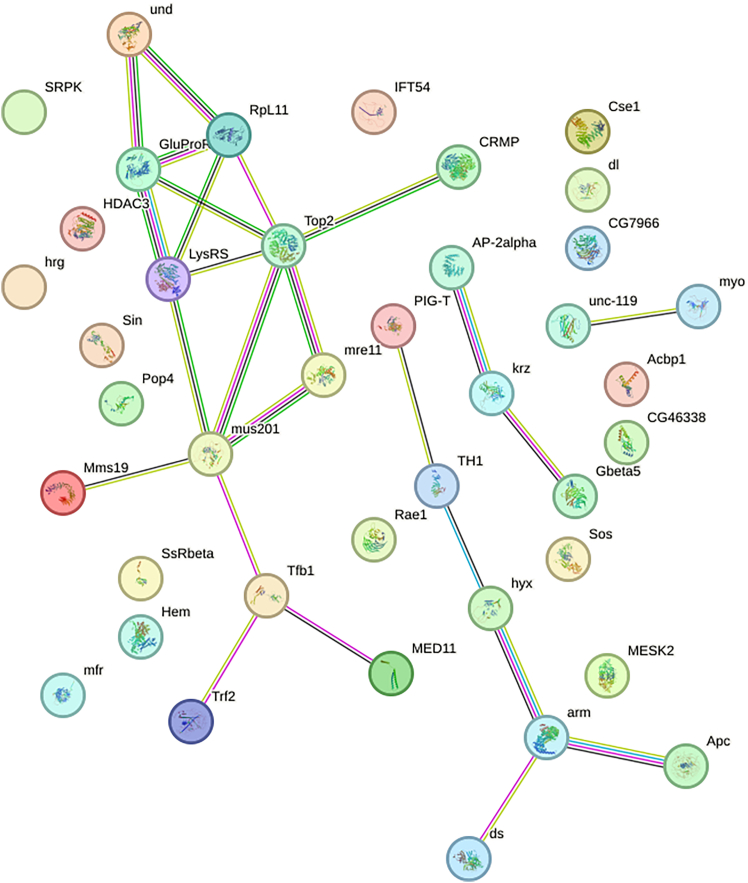
Top 40 human protein samples interaction network

To further validate the biological relevance of these cross-species predictions, we selected the top 40 human protein samples with the highest prediction scores from the independent test. Querying these against the STRING database revealed that 25 of these samples (62.5%) have experimentally supported interactions, as visualized in the interaction network shown in Figure 6. This provides direct biological evidence that the model’s high-confidence predictions are enriched for real interactions, thereby strengthening the practical utility and interpretability of our framework.

## Discussion

### Model advantages and performance analysis

The core contribution of this study is the proposal of SDAERFs—a computational framework integrating a Stacked Denoising Autoencoder with a Random Ferns classifier for the efficient prediction of interactions from protein sequences. The model achieved an accuracy exceeding 98% on both the yeast and human benchmark datasets. Its exceptional performance stems from synergistic optimizations at multiple levels.

First, the effectiveness of feature extraction is foundational to the performance. We leveraged evolutionary information encoded in PSSMs and conducted deep, non-linear feature learning through the SDAE. This process extracts robust high-level representations from variable-length sequences, significantly outperforming traditional dimensionality reduction methods. Second, the choice of classifier is crucial. The Random Ferns classifier achieves a favorable balance between model capacity and regularization due to its unique ensemble structure. Our comparative experiments show that, using the same SDAE-derived features, Random Ferns outperforms common classifiers such as SVM and Random Forest. Most importantly, its superior generalization capability has been independently validated. In a stringent cross-species test, SDAERFs attained an accuracy of 59.24% on unseen human data, significantly higher than the random baseline (50%). This not only rules out severe overfitting on the source data but, more importantly, proves that the model learns evolutionarily conserved interaction patterns rather than dataset-specific noise.

### Computational efficiency and scalability

In practical applications, computational overhead and scalability are key metrics for assessing a model’s utility. The computational load of the SDAERFs framework can be divided into two stages: (1). Generating PSSMs using PSI-BLAST is computationally intensive, requiring seconds to minutes per protein. However, this step is highly parallelizable, and the generated features can be stored and reused for subsequent analyses, constituting a one-time, upfront computational investment. (2). Once PSSM features are obtained, the subsequent SDAE feature extraction and Random Ferns classification are extremely efficient. Prediction for a single protein pair can be completed within milliseconds, demonstrating excellent real-time processing capability.

Compared to many modern deep learning models that rely on large-scale GPUs for both training and inference, SDAERFs offers high performance while presenting a lower barrier to deployment and operational cost. This is particularly advantageous for research scenarios requiring high-throughput screening or operating with limited computational resources. Future optimizations could focus on integrating pre-computed PSSM databases or faster homology search tools to further enhance the overall efficiency of large-scale, proteome-wide predictions.

### Conclusion and future directions

In conclusion, the SDAERFs framework represents an efficient, robust, and interpretable approach for sequence-based PPI prediction by integrating a Stacked Denoising Autoencoder with a Random Ferns classifier on evolutionary PSSM profiles. Its exceptional within-species accuracy, validated generalization across species, and computational efficiency establish it as a valuable tool for the community. Looking forward, this work opens several promising avenues: integrating SDAERFs with state-of-the-art protein language models (e.g., ESM and ProtT5), extending its application to multi-omics and dynamic network prediction across diverse organisms, and ultimately guiding experimental validation to translate computational insights into biological discoveries and therapeutic opportunities.

### Limitations of the study

Although the SDAERFs framework demonstrates robust performance, its current design exhibits certain limitations: the model’s effectiveness is contingent on the quality and evolutionary depth captured by PSSM profiles, which may not adequately represent proteins with few homologs; the feature extraction process, while automated, operates as a comparative “black box,” limiting the interpretability of the specific sequence features deemed critical for interaction; furthermore, the framework relies solely on sequence-derived information, excluding potentially informative structural, functional, or contextual biological knowledge that could enhance prediction accuracy and biological plausibility. Future work will focus on integrating multi-modal data sources and developing more interpretable architectures to address these constraints.

## Resource availability

### Lead contact

Requests for further information and resources should be directed to and will be fulfilled by the lead contact, Zheng Wang (xywangzheng0971@163.com).

### Materials availability

This study did not generate new unique reagents.

### Data and code availability

• The data reported in this article is publicly available on GitHub (https://github.com/wang-devlab/PPI-SDAERFs).
• All original code has been deposited at GitHub and is publicly available as of the date of publication. The DOI is listed in the key resources table.
• Any additional information required to reanalyze the data reported in this article is available from the lead contact upon request.

## Acknowledgments

The authors would like to thank the editors and anonymous reviewers for their constructive comments and suggestions, which have greatly improved the quality of this article. This work was supported by the National Natural Science Foundation of China under Grant 62176146.

## Author contributions

Z.W.: methodology and writing – original draft. Y.L.: project administration, resources, and writing – review and editing. L.W.: funding acquisition, investigation, and writing – review and editing. Z-H.Y.: visualization and writing – original draft. Y-C.L.: formal analysis and writing – original draft. All authors read and approved the final article.

## Declaration of interests

The authors declare no competing interests.

## STAR★Methods

### Key resources table

**Table:** 

REAGENT or RESOURCE	SOURCE	IDENTIFIER
**Deposited data**		

Data and code	This Study	https://github.com/wang-devlab/PPI-SDAERFs

**Software and algorithms**		

Matlab	Open Source	https://www.mathworks.com/products/matlab.html
Python	Open Source	https://www.python.org/

**Other**		

PSI-BLAST:Position-Specific Iterative Basic Local Alignment Search Tool	PSI-BLAST	https://blast.ncbi.nlm.nih.gov/Blast.cgi

### Experimental model and study participant details

This is a computational study. All analyses were performed using publicly available protein-protein interaction benchmark datasets (Yeast and Human). No live experimental models (e.g., animals, plants, microbes, cell lines, primary cultures) or human participants were involved in this research.

### Method details

#### Protein interaction datasets

In this study, we evaluated the effectiveness of the proposed SDAERFs model using two prominent protein-protein interaction datasets derived from the Yeast and Human organisms. The Yeast dataset was obtained from DIP database.^28^ A stringent filtering process was applied, eliminating protein pairs with fewer than 50 residues or those sharing more than 40% sequence identity. This 40% threshold is consistent with standard practices for constructing non-redundant benchmark datasets in yeast PPI prediction. After preprocessing, we obtained 5594 positive interaction pairs. To construct a balanced dataset, an equal number of non-interacting pairs were randomly selected from different subcellular compartments, resulting in a final Yeast dataset of 11,188 pairs.

Similarly, the human data were sourced from HPRD database.^29^^,^^30^ We removed pairs with sequence identity exceeding 25%, which is a widely adopted cutoff in human PPI studies to balance data diversity and the reduction of homology bias. This process identified 3899 validated interacting pairs involving 2502 distinct human proteins. A negative dataset of 4262 pairs was constructed from 661 different proteins, yielding a total Human dataset of 8161 protein pairs.

To summarize, these datasets, prepared using organism-specific filtering criteria common in the field, provide a robust foundation for evaluating the performance of the SDAERFs model across distinct biological contexts.

#### Data matrix of protein sequences

In bioinformatics, the Position-Specific Scoring Matrix (PSSM) is a fundamental and powerful tool for representing the evolutionary conservation patterns of protein sequences. It encapsulates the likelihood of amino acid substitutions at each position of a query sequence, derived from multiple sequence alignments against a large, diverse protein database. This evolutionary profile is highly effective for identifying distantly related homologs and has been widely adopted in numerous sequence-based prediction tasks, including protein-protein interaction prediction.^31^^,^^32^ Formally, for a protein sequence of length L,its corresponding protein PSSM is a matrix of dimensions L×20,and the protein PSSM is defined as follows:

(Equation 1) $${\text{Protein}}_{PSSM}=\left[\begin{array}{cccc} p_{1,1} & p_{1,2} & \cdots & p_{1,20}\\ p_{2,1} & p_{2,2} & \cdots & p_{2,20}\\ \vdots & \vdots & \vdots & \vdots \\ p_{L,1} & p_{L,2} & \cdots & p_{L,20} \end{array}\right]$$

Here, each row corresponds to a specific residue position in the sequence, and each of the 20 columns corresponds to one of the standard amino acids. Each element $p_{i,j}$ in the matrix signifies the likelihood that the *i*th residue will undergo a mutation to the *j*th amino acid throughout the multiple sequence alignments.

To construct the PSSM for each protein in our study, we employed the Position-Specific Iterated BLAST (PSI-BLAST) algorithm.^33^ This iterative search strategy builds a sequence profile by incorporating detected homologs from previous rounds, thereby significantly enhancing sensitivity for detecting remote evolutionary relationships. The search was conducted against the comprehensive and non-redundant SwissProt database^34^ to ensure broad coverage of protein space. Key search parameters were carefully selected to balance sensitivity and specificity: an E-value threshold of 0.001 was used to include statistically significant alignments, and the search was iterated three times to progressively refine the profile and capture more distant homology signals. This process results in a PSSM rich in evolutionary information, which serves as the foundational raw input for our subsequent feature engineering pipeline.

#### Stacked denoising autoencoder feature

To effectively represent each protein, feature extraction is necessary. However, the varying lengths of protein sequences result in PSSM matrices of differing sizes, rendering them unsuitable for direct input into neural networks. To overcome this challenge, this paper uses a fixed-size representation of the PSSM through the use of transposed versions, transforming each PSSM into a matrix with uniform dimensions. The procedure is outlined as follows:

(Equation 2) $$P_{PSSM}^{\prime }={\text{Protein}}_{PSSM}^{T}\times {\text{Protein}}_{PSSM}$$

This $P_{PSSM}^{\prime }$ matrix encapsulates the evolutionary correlation between different amino acid types across the entire protein sequence. Finally, we flatten matrix into a 400-dimensional row vector, which serves as the uniform feature representation for each protein sequence, independent of its original length.

Here, deep and robust features are extracted from the equal-sized PSSM representations of protein sequences by utilizing stacked denoising autoencoder (SDAE) method. The SDAE algorithm is an extension of the basic Autoencoder, designed to handle noisy data and extract hierarchical representations from input data. This section details the architecture and training process of the SDAE used in our model.

An Autoencoder (AE) is a type of neural network used for unsupervised learning, consisting of two main components: an encoder and a decoder. The mathematical operations of AE can be expressed as:

(Equation 3) $$h=f\left(w_{e}x+b_{e}\right)$$

(Equation 4) $$y=g\left(w_{d}h+b_{d}\right)$$

where x denotes the input data, h denotes the latent representation, $w_{e}$ and $w_{d}$ are the weights of the encoder and decoder, respectively, and $b_{e}$ , $b_{d}$ are the biases. The activation functions f(·) and g(·) enable the model to learn non-linear features.

The training objective is to minimize the reconstruction error between the input x and the reconstructed output y, typically measured by the mean squared error:

(Equation 5) $$L=\frac{1}{n}\sum_{i=1}^{n}{\left(x_{i}-y_{i}\right)}^{2}$$

As an effective extension of the basic autoencoder, the Denoising Autoencoder (DAE) introduces a critical enhancement to the AE architecture.^35^^,^^36^ The DAE receives a corrupted version of the data as its input, with the objective of reconstructing the original data. This process prevents the model from merely establishing an identity mapping between the inputs and outputs, thereby capturing more useful information and obtaining valid representations. The DAE comprises an encoder and a decoder, similar to the basic AE. However, prior to the encoding process, the input data x is corrupted into $\hat{x}$ through a stochastic mapping:

(Equation 6) $$\hat{x}=x+\alpha$$

(Equation 7) $$\hat{x}=q_{D}\left(\hat{x}|x\right)$$

The corrupted input $\hat{x}$ is first encoded into a hidden representation *h* through the encoder. Subsequently, the decoder reconstructs the original data from this hidden representation. This training mechanism forces the model to learn robust features that are less sensitive to noise, making the DAE more effective in handling real-world data with inherent noise.

Building on the strengths of the DAE, the Stacked Denoising Autoencoder (SDAE) extends the architecture by stacking multiple DAEs.^37^ This stacking allows the SDAE to learn better descriptors through feeding the outputs of one DAE as the inputs to the next. Each DAE in the stack is trained individually, and their outputs are used as the input for the subsequent layer. This hierarchical structure enables the SDAE to capture progressively higher-level features from the input data.^38^ The training process of the SDAE involves two main phases: (1) Unsupervised Pre-training. Each DAE in the stack is trained in a greedy layer-wise manner. The first DAE is trained on the corrupted input data, and its hidden layer outputs are employed as the input in the next DAE. This procedure is repeated for each subsequent layer. (2) Supervised Fine-tuning. After all layers are pre-trained, the entire network undergoes supervised fine-tuning. A classification layer is added on top of the network to perform the final prediction task. The weights of the network are adjusted using backpropagation to minimize the classification error.

#### Random ferns

Random Ferns (RFs) represent a classification approach inspired by the principles of Random Forests, yet they offer distinct advantages, particularly in scenarios involving high-dimensional data and complex classification tasks.^39^^,^^40^ Here, by giving a candidate patch in an image, our main task can be described as assigning the candidate patch to the most probable class. Let $c_{i},i=0,1\text{,}$ represent the set of classes and $f_{j},j=1,2,...,N\text{,}$ denote the binary features computed on our given candidate patch. Formally, our goal is to find:

(Equation 8) $$\hat{c_{i}}=\underset{c_{i}}{\arg\;\;\max}\;P\left(C=c_{i}|f_{1},f_{2},...,f_{N}\right)$$

where C refers to a random variable associated with the class. Bayes rule is expressed as

(Equation 9) $$P\left(C=c_{i}|f_{1},f_{2},...,f_{N}\right)=\frac{P\left(f_{1},f_{2},...,f_{N}|C=c_{i}\right)P\left(C=c_{i}\right)}{P\left(f_{1},f_{2},...,f_{N}\right)}$$

Suppose there is a uniform prior P(C), by removing the priors $P\left(f_{1},f_{2},...,f_{N}\right)\text{,}$ common to all classes, our problem is equivalent to seeking

(Equation 10) $$\hat{c_{i}}=\underset{c_{i}}{\arg\;\;\max}\;P\left(f_{1},f_{2},...,f_{N}|C=c_{i}\right)$$

According to the Naive Bayesian hypothesis, all features are considered completely independent,

(Equation 11) $$P\left(f_{1},f_{2},...,f_{N}|C=c_{i}\right)=\prod_{j=1}^{N}P\left(f_{j}|C=c_{i}\right)$$

The assumption of independence is usually inaccurate and unreliable. In addition, this will result in underestimating the true posterior probabilities. To deal with complex problems and make them easier to solve, an effective method is proposed, which is the features of the ferns that were divided into M groups. Its size is S=N/M and these groups are defined as the number of ferns. Therefore, the joint probability of each fern features can be calculated. The formula for the conditional probability is presented in the following manner:

(Equation 12) $$P\left(f_{1},f_{2},...,f_{N}|C=c_{i}\right)=\prod_{k=1}^{M}P\left(F_{k}|C=c_{i}\right)$$

where $F_{k}=\left\{f_{\rho_{\left(k,1\right)}},f_{\rho_{\left(k,2\right)}},...,f_{\rho_{\left(k,S\right)}}\right\},k=1,2,\cdots M\text{,}$ refers to the kth ferns and ρ(k,j) represents a random permutation function. In addition, the conditional probabilities for each fern and class can be computed. For each fern $F_{m}\text{,}$ these conditions can be expressed below.

(Equation 13) $$p_{k,c_{i}}=P\left(F_{m}=k|C=c_{i}\right)=\frac{N_{k,c_{i}}}{N_{c_{i}}}$$

where $N_{k,c_{i}}$ represents the total number of training instances for features $F_{m}=k$ of class $c_{i}$. The total number of instances corresponding to class $c_{i}$ is denoted as $N_{c_{i}}$. If the number of instances is not infinitely large, $N_{k,c_{i}}$ and $p_{k,c_{i}}$ may be 0. So here define $p_{k,c_{i}}$ as:

(Equation 14) $$p_{k,c_{i}}=\frac{N_{r}+N_{k,c_{i}}}{K\times N_{r}+N_{c_{i}}}$$

where $N_{r}$ is the regularization term. In our experiment, we configured two key parameters for the random ferns classifier: the depth parameter *S* was set to 20, and the number of ferns *M* was configured to 50. The attributes extracted by the SDAE deep learning method were input into the RFs classifier to predict interactions between protein pairs.

#### Evaluation indicators

To comprehensively evaluate the predictive performance of the SDAERFs model in predicting PPIs, we employed a robust set of evaluation metrics. These metrics were carefully selected to ensure a thorough assessment of the model’s accuracy, reliability, and generalizability. The evaluation process utilized the five-fold cross-validation technique to minimize over-fitting and ensure the robustness of the predictive ability of SDAERFs. The five-fold cross-validation involved randomly dividing the entire dataset into five subsets of approximately equal size. The final metrics were computed as the average and standard deviation across all iterations, providing a reliable estimate of the model’s predictive capabilities.^27^ The evaluation metrics used in this study included accuracy (ACC), sensitivity (SN), precision (PE), and Matthews correlation coefficient (MCC). The definitions and formulas for these metrics are as follows:

(Equation 15) $$ACC=\frac{TP+TN}{TP+TN+FP+FN}$$

(Equation 16) $$SN=\frac{TP}{TP+FN}$$

(Equation 17) $$PE=\frac{TP}{TP+FP}$$

(Equation 18) $$MCC=\frac{TN\times TP-FN\times FP}{\sqrt{\left(TP+FP\right)\times \left(TN+FP\right)\times \left(TN+FN\right)\times \left(FN+TP\right)}}$$

where *TP,TN,FP* and *FN* represent True Positive, True Negative, False Positive and False Negative, respectively. Along with these metrics, we generated ROC curves and computed the AUC^41^ to provide a graphical and quantitative assessment of the model’s predictive power. By employing these evaluation metrics, we ensured a comprehensive and objective assessment of the SDAERFs model, enabling a detailed understanding of its strengths and limitations in predicting PPIs.

### Quantification and statistical analysis

In the final evaluation of the model’s performance, we employed a 5-fold cross-validation approach. The ultimate results consisted of the mean of the 5-folds, with specific evaluation metrics including ACC, SN, PE,MCC and AUC.
